# Dynamic Causal Modelling of the Reduced Habituation to Painful Stimuli in Migraine: An EEG Study

**DOI:** 10.3390/brainsci10100712

**Published:** 2020-10-07

**Authors:** Iege Bassez, Frederik Van de Steen, Katia Ricci, Eleonora Vecchio, Eleonora Gentile, Daniele Marinazzo, Marina de Tommaso

**Affiliations:** 1 Department of Data Analysis, Ghent University, Henri Dunantlaan 1, 9000 Gent, Belgium; frederik.vandesteen@ugent.be (F.V.d.S.); Daniele.Marinazzo@UGent.be (D.M.); 2 Applied Neurophysiology and Pain Unit, Department of Basic Medical Sciences, Neurosciences and Sensory Organs, Bari Aldo Moro University, Piazza Giulio Cesare 11, 70123 Bari, Italy; katiari86@gmail.com (K.R.); Eleonora.Vecchio@gmail.com (E.V.); eleonora.gentile.psico@gmail.com (E.G.)

**Keywords:** migraine, EEG, LEP, habituation, pain, DCM, connectivity

## Abstract

A consistent finding in migraine is reduced cortical habituation to repetitive sensory stimuli. This study investigated brain dynamics underlying the atypical habituation to painful stimuli in interictal migraine. We investigated modulations in effective connectivity between the sources of laser evoked potentials (LEPs) from a first to final block of trigeminal LEPs using dynamic causal modelling (DCM) in a group of 23 migraine patients and 20 controls. Additionally, we looked whether the strength of dynamical connections in the migrainous brain is initially different. The examined network consisted of the secondary somatosensory areas (lS2, rS2), insulae (lIns, rIns), anterior cingulate cortex (ACC), contralateral primary somatosensory cortex (lS1), and a hidden source assumed to represent the thalamus. Results suggest that migraine patients show initially heightened communication between lS1 and the thalamus, in both directions. After repetitive stimulations, connection strengths from the thalamus to all somatosensory areas habituated in controls whereas this was not apparent in migraine. Together with further abnormalities in initial connectivity strengths and modulations between the thalamus and the insulae, these results are in line with altered thalamo-cortical network dynamics in migraine. Group differences in connectivity from and to the insulae including interhemispheric connections, suggests an important role of the insulae.

## 1. Introduction

When the brain receives repetitive sensory stimulations, it will gradually decrease its evoked activity, which is a phenomenon known as habituation. Impaired habituation characterizes the interictal brain of migraine patients as it is a consistent finding in response to repetitive stimuli from several sensory modalities [1,2]. Proposed mechanisms that could underlie this atypical habituation to sensory stimuli in interictal migraine are increased cortical excitability, decreased inhibition and decreased preactivation levels [1,3]. Lower amplitudes in the first few trials of evoked potentials in migraine compared to healthy controls could be explained by reduced preactivation levels of the sensory cortices. These reduced preactivation levels may be followed by deficient inhibition, resulting in a hyper-responsivity to repetitive stimulations [1].

Reduced cortical habituation during the interictal period has also been found when migraine patients repeatedly receive painful stimulations [4,5,6,7,8]. By recording EEG data while administering several painful laser stimuli, this reduced habituation becomes visible in the N2P2 amplitude responses at the vertex [5,6,7,8]. In a functional magnetic resonance imaging (fMRI) study that used repeated stimulations, migraine patients showed increases in pain ratings which were accompanied with increases in neural activity in the bilateral anterior insula, the midcingulate cortex and the thalamus, while the control group showed decreases both in ratings and neural activity in these brain regions [4]. The same study also investigated habituation to repeated olfactory stimulations, which was not impaired in migraine. Given that olfactory input is not relayed in the thalamus and that increases were found in the thalamus, insulae, and cingulate cortex, the authors suggest that a thalamo-cortical network might be responsible for the impaired habituation.

A study on brain connectivity showed that interictal migraine patients have different cortical connection patterns than healthy controls during painful stimulation [8]. Migraine patients showed higher synchronization between the right temporal-central-frontal and posterior parietal areas whereas healthy control participants showed increased synchronization between the central and frontal cortical and temporal-parietal areas. In addition to functional connectivity, effective connectivity results showed that during laser stimulation migraine patients’ activity measures around two centro-parietal channels were more connected with almost all the other scalp channels activations compared to healthy controls. The authors suggest that although no inferences can be made about connectivity in cortical regions, the centro-parietal network that was more effectively connected in migraine could possibly reflect the laser-evoked potentials (LEPs) sources that are first recruited, which are the bilateral secondary somatosensory areas and the insulae. Importantly, strength of cortical N2P2 habituation to painful laser stimuli was negatively correlated with the averaged effective connectivity results, suggesting the possibility that the impaired habituation found in migraine patients might be the result of increased cortical connectivity within the pain network. As this study only looked at connectivity at the scalp level, it still remains unknown how brain regions are connected and modulated during repeated painful stimulations. Given that the generators of LEPs are the bilateral insula (Ins), the bilateral secondary somatosensory regions (S2), the anterior cingulate cortex (ACC), and the contralateral somatosensory region (S1) [9], it is likely that connections between these regions as well as between these regions and the thalamus, determine the habituation pattern.

In the current study, we therefore investigated effective connectivity patterns between these regions. Specifically, we used dynamic causal modelling (DCM) to investigate the modulations in effective connectivity from a first to final block of trigeminal LEPs. Our goals were (1) to gain insight in the neural mechanisms underlying impaired interictal habituation to pain by comparing the modulations in effective connectivity between healthy controls and migraine patients and (2) to see if the interictal brains of migraine patients are more or less connected in the initial block of trials compared to healthy controls.

## 2. Materials and Methods

### 2.1. Participants

Twenty-three migraine patients without aura (16 females, M age = 35.13 years, SD age = 12.57 years) participated in this study. Patients were diagnosed according to ICHD-3 criteria [10] and diagnoses were confirmed considering more recent criteria [11]. The migraine patients in this study had a history of migraine attacks between 2 and 30 years (M = 13.22 years, SD = 8.05 years). The mean headache frequency was 6.65 days with headache in a month (SD = 4.38). The headache intensity varied from 6 to 10 with a mean of 9 (SD = 1.30) on a scale of 10, indicating that most patients experienced severe painful migraine attacks. Patients were tested between headache attacks, at least 72 h after the last attack, and more than 48 h before the next one. This was ascertained by direct or telephone contact. The patients were tested during a first visit to the clinic and therefore were not yet prescribed any drugs. Twenty healthy volunteers (13 females, M age = 33.05 years, SD age = 12.57 years) were selected based on the absence of personal and first-degree familiar history of migraine. All participants gave their informed consent for inclusion before they participated in the study. The study was conducted in accordance with the Declaration of Helsinki, and the protocol for this neurophysiological study was approved on the 31 January 2018 by the Ethical Committee of Bari Policlinic General Hospital.

### 2.2. EEG and Procedures

The patients laid on a couch with their eyes open in a warm semi-darkened room. A 61 channels montage was used. The recording electrodes were placed on the scalp referred to the nasion, according to the extended International 10–20 System. The recording system was a MICROMED EEG apparatus (Micromed Brain Quick, Mogliano Veneto, Italy). Two additional electrodes were positioned below the eyes for electrooculogram recording. The impedance was kept below 4 KΩ. During the recording session, digital filters in the 0.1–70 Hz range and a 50 Hz notch filter were applied to allow signal inspection. Participants received a series of 15 painful laser stimulations on the right forehead, corresponding to the first branch of the trigeminal nerve. The inter stimulation interval was self-paced and varied around ±10 s. The laser stimulations were cutaneous heat stimuli delivered by a CO_2_ laser (wavelength: 10.6 mm; beam diameter: 2 mm; ELEN, Florence, Italy). To avoid damage to the skin, fatigue, or sensitization of nociceptors, the irradiated spot was shifted after each stimulus. Before each series, the intensity (min 6 Watt, max 9 Watt) and duration (min 15 ms, max 45 ms) of the laser stimulations were adjusted so that the participants genuinely experienced the laser stimulations as painful. When the participants rated the stimuli as a pinprick (pain threshold), the power was increased with one unit. After the series of laser stimuli, patients were asked to indicate the perceived pain during that series on a visual analogue scale (VAS) ranging from 0 to 100. On the VAS, the white color corresponding to 0 indicated no pain sensation while the intense red corresponding to 100 indicated the worst pain conceivable.

### 2.3. Preprocessing EEG

The data were preprocessed in MATLAB with an automatic pipeline using EEGLAB (v14.1.1) [12] and plug-in functions. The data were bandpass filtered between 1 and 40 Hz using a Hamming windowed sinc FIR filter with filter order 846 (calculated using the default heuristic in the pop_eegfiltnew function). We then applied the Artifact Subspace Reconstruction method (ASR, clean_rawdata plugin for EEGLAB) to correct continuous data and reject bad channels and data segments [13,14]. The remaining data were re-referenced to the average. Independent component analysis (ICA) was then performed where artefactual components were automatically removed by using a machine learning algorithm named Multiple Artifact Rejection Algorithm (MARA) [15]. Components with a “probability of being artefactual” higher than 0.90 were removed. The data were then epoched in the time interval −0.5 to 1 s and the baseline was removed.

### 2.4. N2P2 Amplitudes at the Cz Channel

To see if we could replicate the habituation deficit in migraine in response to repetitive laser stimuli, we calculated the habituation index (HI) at the Cz channel as described in previous studies [8,16]. Specifically, the 15 laser trials were divided in three blocks of five trials and the percentage change in N2P2 amplitudes (peak-to-peak) from the first to third block was calculated ((first block–third block)/first block ×100). Reductions in N2P2 amplitudes (i.e., habituation) from the first to third block correspond to positive HI values while facilitations in N2P2 amplitudes correspond to negative HI values. A nonparametric permutation two-sample *t*-test was used to compare the habituation indices between groups.

In addition, we investigated whether the N2P2 amplitudes (peak-to-peak) at the Cz channel in the first block were lower in the migraine group by using a nonparametric permutation two-sample *t*-test.

### 2.5. Dynamic Causal Modelling

The average LEPs (61 channels with 206 timeframes between 0 and 800 ms) from the first and third block were imported in SPM12 (Wellcome Trust Centre for Human Neuroimaging) running on MATLAB (version 2017b). We investigated effective connectivity strengths between the sources of LEPs in the first block and modulations in effective connectivity from a first to final block using dynamic causal modelling (DCM) for evoked responses [17]. In short, DCM tries to explain how event-related potentials and their modulations are generated by brain dynamics, using a biologically plausible generative model (neural model + forward model). Posterior estimates of connectivity strengths and modulations in connectivity (amongst other parameters) can be obtained by inverting the generative model. The generative model is inverted by using a variational Bayesian optimization scheme that uses free energy—a lower bound on the log model evidence—as the objective function [18].

**Figure 1:**
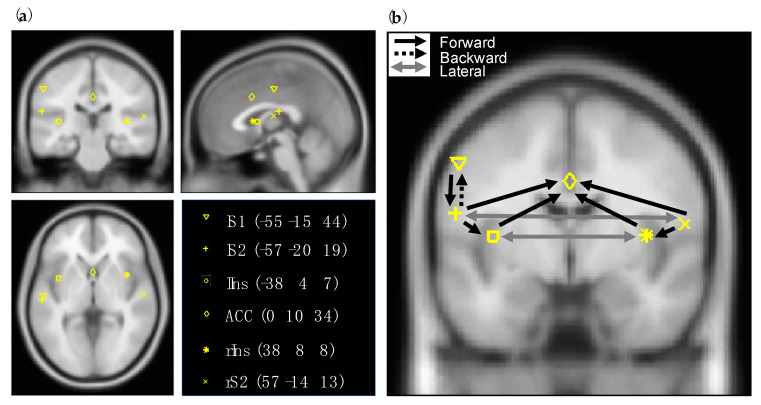
The regions and connectivity pattern between them as specified in the dynamic causal modelling (DCM) analysis. (**a**) Prior MNI coordinates of the laser-evoked potentials (LEPs) sources. (**b**) Forward, backward and lateral connections that were estimated with DCM. Note that for visibility reasons the forward connections from the hidden source (thalamus) to all the regions and backward connections from those regions to the hidden source are not displayed in the figure but were estimated.

As neural model, the ‘ERP’ convolution-based neural mass model was used [19]. In this model, each source (i.e., brain region) has three cell subpopulations, comprising excitatory spiny stellate cells, inhibitory interneurons, and excitatory pyramidal cells. Extrinsic connections between regions can be either forward, backward, or lateral depending on the target and seed neuronal subpopulation. Based on the hierarchical organization of the cortex, the type of connection can be inferred [20]. The regions and prior MNI (Montreal Neurological Institute) coordinates were based on the review of Garcia-Larrea et al. (2003) on the generators of LEPs [9]. Chosen coordinates where checked with Neurosynth where we looked at term-based meta-analyses [21]. The network consisted of the contralateral primary somatosensory cortex (lS1), the secondary somatosensory areas (lS2, rS2), the insular regions (lIns, rIns), the anterior cingulate cortex (ACC), and a hidden source assumed to represent the thalamus. By hidden, we mean a region that is assumed not to contribute to the observed signals directly [22]. The connectivity pattern between the regions was determined based on Price [23] and May [24]. Forward connections were specified according to ascending pain pathways. Backward connections were specified from all regions to the hidden source and from lS2 to lS1. Hemispheres were connected with lateral connections. The prior MNI coordinates of the regions and presumed coupling between them can be found in Figure 1. The source locations were optimized in the DCM analysis.

For the spatial forward model, the equivalent current dipole method was used (‘ECD’ option in SPM12). Canonical T1 images and the boundary element method (BEM) were used to obtain leadfield matrices.

### 2.6. Parametric Empirical Bayes

Connectivity strengths in the first block and modulations in connectivity strengths over blocks as estimated with DCM were compared between groups using parametric empirical Bayes (PEB) [25], which is a Bayesian linear model where the dependent variables are the vectorized DCM parameters. The design matrix contained a column of ones and a column of dummy variables with zeros representing the control group and ones representing the migraine group. With PEB, uncertainties of the estimated connectivity strengths and connectivity modulations are taken into account by the posterior covariance. Bayesian model reduction (BMR) and a greedy search was used to remove redundant parameters from the full model [25,26]. Parameters with a posterior probability of being different from zero higher than 0.99 were interpreted.

## 3. Results

**Figure 2:**
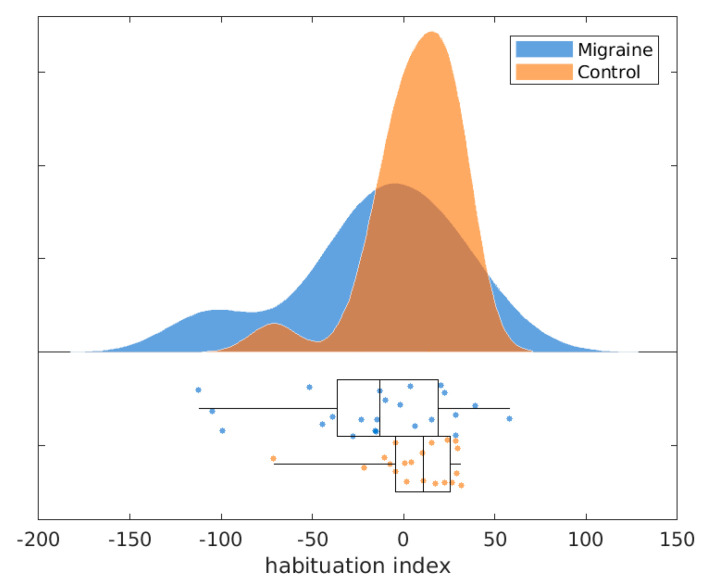
Habituation indices for migraine patients and healthy controls.

The pain ratings were slightly higher in the migraine group (M = 65.52, SD = 18.90) than in the control group (M = 58.40, SD = 21.49) but this difference was not statistically significant (t = 1.15, *p* = 0.26). As in previous studies, we found that the HIs of the N2P2 amplitudes at the vertex (see Figure 2) were lower in the migraine group (M = −15.24, SD = 44.87) than in the control group (M = 6.63, SD = 24.05), this difference was statistically significant (t = −2.03, *p* = 0.02). The N2P2 amplitudes in the first block were significantly lower in the migraine group (M = 11.44, SD = 6.13) compared to the control group (M = 15.04, SD = 7.00; t = −1.78, *p* = 0.04).

**Figure 3:**
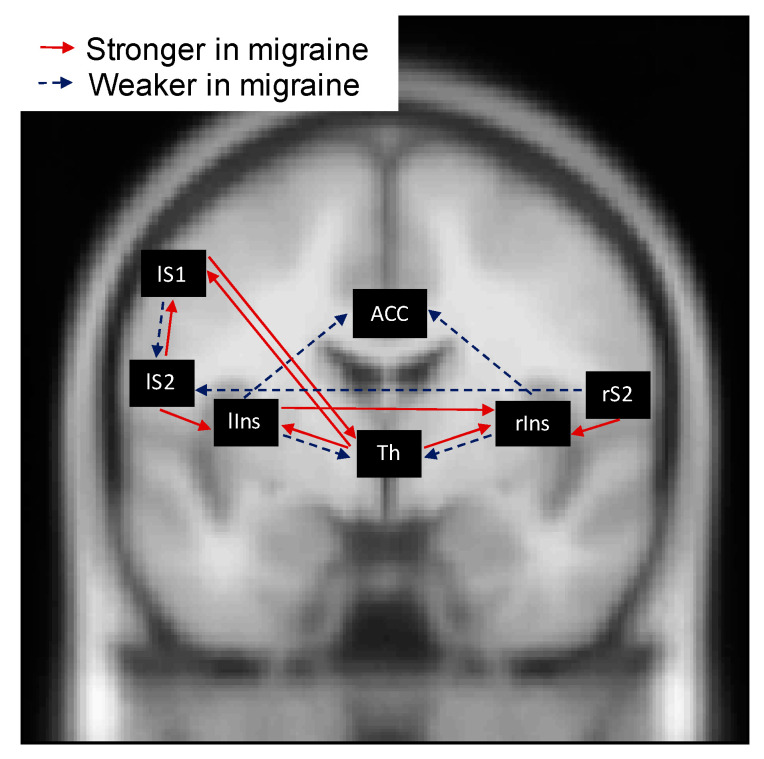
Parametric empirical Bayes results on the connectivity strengths in the first block as estimated with dynamic causal modelling. Only parameters with a posterior probability of being different from zero >0.99 are visualized. Red arrows (solid arrows) indicate that the connectivity strength is stronger in the migraine group, while blue arrows (dashed arrows) indicate that the connectivity strength is weaker in the migraine group. For precise differences between groups, see main text.

PEB results comparing groups on connectivity strengths in the first block, showed that following forward connections were initially stronger in the migraine group compared to the control group (see Figure 3): the connections from the hidden source to both the rIns (β1 = 0.33, posterior SD β1 = 0.10) and the lIns (β1 = 0.47, posterior SD β1 = 0.09) as well as to the lS1 (β1 = 0.22, posterior SD β1 = 0.09), the connection from the rS2 to the rIns (β1 = 0.72, posterior *SD* β1 = 0.12) and from the lS2 to the lIns (β1 = 0.40, posterior SD β1 = 0.12). The following forward connections were weaker in the migraine group compared to the control group: the connection from the rIns to the ACC (β1 = −0.80, posterior SD β1 = 0.10), from the lIns to the ACC (β1 = −0.18, posterior *SD* β1 = 0.06) and from the lS1 to the lS2 (β1 = −0.31, posterior SD β1 = 0.11).

The following backward connections were initially stronger in the migraine group compared to the control group (see Figure 3): the connection from the lS2 to the lS1 (β1 = 0.29, posterior SD β1 = 0.07) and from the lS1 to the hidden source (β1 = 0.45, posterior SD β1 = 0.08). The following backward connections were weaker in the migraine group compared to the control group: the connection from the rIns to the hidden source (β1 = −0.44, posterior *SD* β1 = 0.11) and from the lIns to the hidden source (β1 = −0.43, posterior *SD* β1 = 0.14).

PEB results comparing groups on connectivity strengths in the first block showed that the *lateral* connectivity from the lIns to the rIns (β1 = 0.37, posterior SD β1 = 0.11) was stronger in the migraine group compared to the control group while the lateral connectivity from the rS2 to the lS2 was weaker in migraine compared to control (β1 = −0.61, posterior SD β1 = 0.11) (see Figure 3).

**Figure 4:**
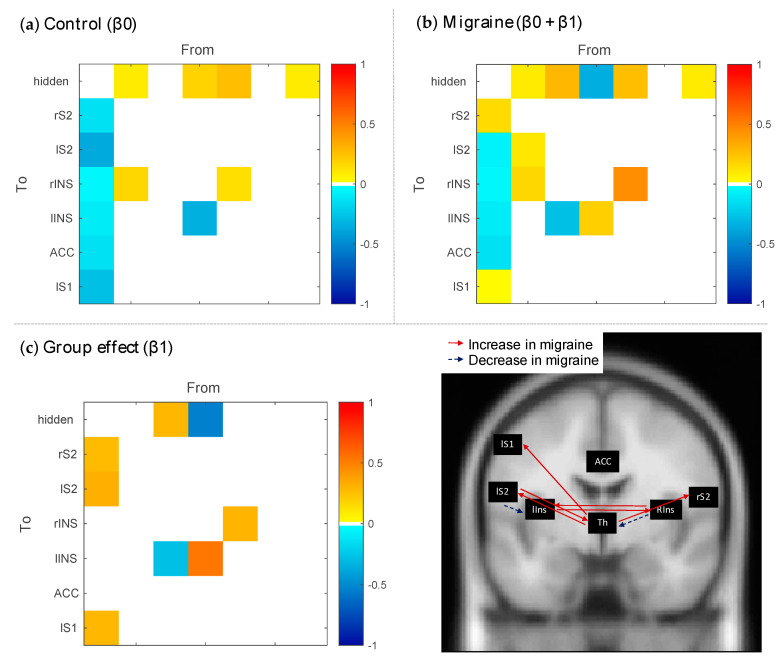
Parametric empirical Bayes results comparing connectivity modulations between groups. (**a**) Group mean modulation of connectivity for the control group. (**b**) Group mean modulation of connectivity for the migraine group. (**c**) Connectivity modulations that show a group effect. Only parameters with a posterior probability of being different from zero >0.99 are colored in the left panel and visualized as arrows on an anatomical scan in the right panel.

PEB results comparing connectivity modulations between groups (see Figure 4) showed that the control group had decreases in connection strengths in the final block compared to the first block from the hidden source to the rS2 (β0 = −0.13, posterior SD β0 = 0.08), the lS2 (β0 = −0.37, posterior SD β0 = 0.06), and the lS1 (β0 = −0.27, posterior *SD* β0 = 0.06) while the migraine group showed an increase (β0 + β1 = 0.14, posterior SD β1 = 0.10), a negligible decrease (β0 + β1 = −0.05, posterior SD β1 = 0.08), and a negligible increase (β0 + β1 = 0.02, posterior SD β1 = 0.08), respectively. Further, there was an increase in connectivity from the rIns to the hidden source for the controls (β0 = 0.19, posterior SD β0 = 0.10), while this connection decreased over blocks in migraineurs (β0 + β1 = −0.35, posterior *SD* β1 = 0.13). An increase in connectivity from the lS2 to the hidden source (β0 + β1 = 0.28, posterior *SD* β1 = 0.08), and a decrease from the lS2 to the lIns (β0 + β1 = −0.28, posterior *SD* β1 = 0.06), was visible for migraineurs but not for controls (β0 = 0 for both). Finally, lateral connections between insular regions were more increased in migraine (β0 + β1 = 0.44 for lIns > rIns, posterior *SD* β1 = 0.11 and β0 + β1 = 0.19 for rIns > lIns, posterior *SD* β1 = 0.14) compared to controls (β0 = 0.14 for lIns > rIns, posterior *SD* β0 = 0.09 and β0 = −0.34 for rIns > lIns, posterior *SD* β0 = 0.11).

## 4. Discussion

In this study we investigated (1) whether the interictal brains of migraine patients are more or less connected in the initial block of trigeminal laser stimulations compared to healthy controls and (2) which cortical effective connectivity modulations underlie habituation in healthy controls and how this differs for migraine patients who show reduced habituation. We used DCM to estimate effective connectivity strengths and modulations in connectivity strengths between the brain generators of LEPs and then used PEB to compare groups.

We will first discuss the thalamo-cortical connections and then continue with the cortico-cortical connections. The initial connectivity strengths from the hidden source (presumed to be the thalamus) to the secondary somatosensory cortices did not differ significantly between groups. However, the initial connectivity strength from the thalamus to the contralateral primary somatosensory cortex and the backward connection strength from this region to the thalamus were increased in the migraine group suggesting an initial heightened communication between these regions in migraine. Note that we cannot distinguish whether the backward connection from S1 has an excitatory or inhibitory effect (excitatory connection to inhibitory interneurons) on thalamic activity. For the modulations in connectivity strengths from the first to final block, we found reduced connection strengths from the thalamus to all the somatosensory regions for the control group while this was not apparent in the migraine group. This suggests that under repetitive stimulation, connection strengths from the thalamus to somatosensory areas habituate (i.e., connections strengths become weaker) in healthy individuals whereas this is not the case in migraine. The backward connectivity strength from the lS2 to the thalamus stayed constant in the control group while it increased after repetitive stimulation in the migraine group.

The connectivity pattern between the thalamus and insulae was also different in migraine patients as compared to controls. Initially in the first block, the connectivity strengths from the thalamus to the left and right insulae were increased in the migraine group compared to controls, while the backward connectivity strengths from the thalamus to the bilateral insulae were decreased. This could possibly be a reduction of connectivity that has an inhibitory effect. The connectivity strength from the rIns to the thalamus became even more decreased during the last block of LEPs in the migraine group, while this connection increased over blocks in the control group. If the insula had an inhibitory effect on thalamic activity, then this mechanism might be impaired in migraine. Under the assumption that the hidden source indeed represents the thalamus, these thalamo-insular and thalamo-somatosensory connectivity results are in line with atypical thalamo-cortical network dynamics in migraine with a complex interplay between regions. It is not surprising that these pathways are altered here as they are involved in migraine pathophysiology; insular and somatosensory regions also receive nociceptive input from the thalamus originating from the dura [27] and are part of the trigeminovascular system [28].

For the cortico-cortical connections between somatosensory regions, we saw that in the initial block, the connectivity strengths from lS1 to lS2 and from rS2 to lS2 were weaker in the migraine group than in the control group. On the other hand, the backward connectivity from the lS2 to the lS1 was initially stronger in the migraine group. The mix of increased and decreased connectivity between these regions suggest an altered cross-talk between somatosensory regions in migraine.

Both the left and right S2 showed higher initial connectivity strengths towards the right and left insula in the migraine group. The insula then forwards nociceptive information to the ACC [23], these connectivity strengths were initially decreased in migraine. The initial lower forward connectivity strengths from the insulae to the ACC likely resulted in less ACC activity in the migraine group and may thus potentially explain the lower N2P2 amplitudes in the first block as the ACC is one of the sources giving rise to this LEP response [9]. The lateral connectivity strength from the lIns to the rIns was also initially higher in the migraine group. Lateral connectivity strengths between the insulae further increased over blocks in the migraine group. This increased interhemispheric insulae connectivity together with the increased connectivity from the thalamus to the insulae might be in accordance with the results of Stankewitz et al. (2013) who found increased neural activity in the bilateral insula over a repetitive painful stimulation session [4]. Over blocks, the connectivity strength from the lS2 to lIns (which was initially higher in migraine compared to controls) did decrease in migraine while it stayed constant over blocks in the control group. Overall, many differences between migraine and controls were found in connectivity from and to the insulae, including thalamo-insular connections, suggesting an important role of the bilateral insula. In accordance with this, it was recently reviewed that the insula shows functional and structural changes in migraine, which corresponds with the knowledge that this region is involved in many processes that are altered in migraine (for a review see [29]). These altered processes include amongst others pain processing, vestibular function, and autonomic function. It has been shown that trigeminovascular, vestibular, and visceral inputs project from the thalamus to the insula [27,30,31,32]. In the context of pain, the insula is referred to “a multidimensional integration site for pain” [33]. Due to its bidirectional dense connectivity profile, the insula receives and sends important information. By examining patients with insular lesions, Starr et al., (2009) suggested that “the insula may be importantly involved in tuning cortical regions to appropriately use previous cognitive information during affective processing” (p. 2684) [34]. The complex interplay between the insula and the other regions found here, might reflect this integrating-tuning process and how it is altered in migraine.

A recent study examining the propensity that individuals experience increasing pain with repeated painful stimulations, showed that this might reflect stronger connectivity in the ascending pathway, namely the pathway from the thalamus to the S1, and weaker connectivity in the descending pain-modulatory pathway [35]. In this study we found that migraine patients, who are characterized by impaired habituation to repeated painful stimulations, also show heightened communication between the thalamus and S1. In general, the thalamo-somatosensory interactions were increased in migraine. De Tommaso et al. (2015) also suggested that the impaired habituation found in migraine patients might be the result of increased cortical connectivity within the pain network [8]. Our results also lean to this conclusion as most connectivity results showed increases in migraine compared to controls. However, the complete picture might be more complicated as we also found pathways that showed reduced connectivity in migraine. Future research could investigate this further by distinguishing backward connections with an inhibitory effect from those with an excitatory effect.

## 5. Conclusions

Our results indicate that reduced habituation to pain generally corresponds to impaired dynamics of cortical and thalamo-cortical connections in migraine patients. The altered functioning of the thalamo-cortical and cortico-cortical network under repetitive experimental phasic nociceptive stimulation could confirm a basic abnormality in pain processing. Further studies could clarify how this dysfunction could predispose migraine evolution into chronic disease, and how it could be reverted by acute and preventive treatments.
